# Effect of COVID-19 pandemic on serious mental illness-related outpatient department utilization in Ningbo, China: an interrupted time series analysis

**DOI:** 10.3389/fpsyt.2023.1199408

**Published:** 2023-07-13

**Authors:** Lian Li, Hongying Yang, Rongxiang Zhang, Yucheng Wang, Guolin Bian

**Affiliations:** Public Health Department, Ningbo Kangning Hospital & Affiliated Mental Health Centre, Ningbo University, Ningbo, Zhejiang, China

**Keywords:** COVID-19, interrupted time series analysis, serious mental illness, outpatient department utilization, Ningbo

## Abstract

**Background:**

Globally, the coronavirus disease 2019 (COVID-19) pandemic has negatively affected mental health services, but there is no clear evidence of this in China. Therefore, we examined the effect of the COVID-19 pandemic on the use of serious mental illness (SMI)-related outpatient services in Ningbo, China.

**Methods:**

We analyzed the trends in monthly SMI-related outpatient department utilization from January 2018 to June 2022 using interrupted time series (ITS) regression analysis, and we defined the onset of the COVID-19 pandemic as January 2020. We also performed ITS regression analyses for sex and age subgroups.

**Results:**

A significant difference in the monthly number of outpatient visit slopes before and after the onset of the pandemic was shown in the SMI analysis [−175.6, 95% confidence interval (CI) (−338.3 to −12.9), *p* < 0.05]. All sex and age categories, except the 20–30 years age category, showed statistically significant changes in their slopes after the onset of the pandemic. Significant differences in the number of outpatient visit slopes before and after the onset of the pandemic were seen for schizophrenia and bipolar disorders [−153.3, 95% CI (−294.1 to −12.5) and −16.8 (−31.0 to −2.6), respectively]. Moreover, a negative relationship was observed between the monthly number of outpatient visits and the number of incidents and accidents due to SMI (*r* = −0.38, *p* < 0.05).

**Conclusion:**

The COVID-19 pandemic has had a negative effect on SMI-related outpatient visits in Ningbo, especially by patients with schizophrenia. A strategy should be developed and implemented to maintain access to SMI services during the COVID-19 pandemic.

## 1. Introduction

Globally, more than 970.1 million people suffered from mental illness in 2019 (1), corresponding to an increase of 48% over the past 30 years. There were 39.5 million patients with bipolar disorder and 23.6 million with schizophrenia globally in 2019 (1). In China, the lifetime prevalence of mental disorders is also very high, and was approximately 16.6% in 2018 (2), resulting in a very high disease burden. Mental illness, especially severe mental illness (SMI, e.g., schizophrenia and bipolar disorder), requires continuous treatment (3, 4). Long-term rehabilitation with community care and maintaining antipsychotic treatment are essential to improve negative psychotic symptoms and social dysfunction and prevent relapse in patients with SMI (5, 6). Thus, accessible, timely, and adequate mental healthcare is a prerequisite for the rehabilitation of patients with SMI (5). However, mental healthcare in many countries is currently confronted with challenges (7, 8).

The coronavirus disease 2019 (COVID-19) pandemic has been a major public health event and has contributed to major changes to people’s daily lives (9–11). To contain the spread of COVID-19, most countries have adopted strict public health measures, including requiring the wearing of face masks in public places, restricting social gathering activities, and quarantining those in close contact with patients (12, 13). Given the persistence of the COVID-19 pandemic, mental illness is now more frequent and more serious in both previously unaffected people and patients with mental illness (14). Moreover, the pandemic resulted in an increased demand for mental health services (14–16). For example, some mental health institutes temporarily suspended their services due to local outbreaks of COVID-19 (12), and certain strict public health measures made it difficult for patients with SMI to access mental healthcare (17). Meanwhile, the fear of contracting COVID-19 also restricted people’s daily activities, including their willingness and ability to visit hospitals (17).

Many studies performed in high-income countries have confirmed the negative effect of the COVID-19 outbreak on the use of mental health services (7, 8, 18). However, no such study has been conducted in China, which has a large number of patients with mental illness (2). Moreover, there are no data on the effect of the onset of the pandemic on different types of SMI-related outpatient visits. Therefore, this interrupted time-series (ITS) analysis investigated whether the COVID-19 pandemic decreased SMI-related outpatient department utilization in Ningbo, China from January 2018 to June 2022.

## 2. Methods

### 2.1. Study design and population

There are six districts and four counties in Ningbo. With a population of about 9.6 million in 2022, Ningbo is one of the largest cities in the Yangtze River Delta region on the southeast coast of China. When local outbreaks happened in Ningbo, unprecedented rigorous public health interventions were implemented in the areas of outbreak (from communities to districts), including quarantines, stay-at-home orders, outpatient department at the hospitals were closed and travel restrictions. We used ITS analysis to investigate whether the COVID-19 pandemic affected SMI-related outpatient department utilization in Ningbo. All outpatient treatments for SMI were collected retrospectively each month through the electronic health record (EHR) system. EHR system included outpatient medical information of all specialized and general hospitals, and community hospitals in Ningbo. Date of hospital visits, diagnosis of disease and International Classification of Diseases, Revision 10 (ICD-10) in EHR system was used for our analysis. SMIs included schizophrenia, bipolar disorders, and others (such as schizoaffective disorders, epileptic psychosis, intellectual disabilities, and delusional disorders). All SMIs included in our study were defined based on the ICD-10 code, and the details are shown in Supplementary Table S1. Finally, SMI data were collected from January 2018 to June 2022. The onset of the COVID-19 pandemic was defined as January 2020, which is when it first affected Ningbo. The sex and age of each patient were also collected in our study. This study was approved by the Ethics Committee of Ningbo Kangning Hospital, and permission was granted to analyze the data (NBKNYY-2022LC-41).

### 2.2. Outcomes

The primary outcomes were the immediate and long-term effects of the COVID-19 pandemic on SMI-related outpatient visits. The secondary outcomes were the effect of the COVID-19 pandemic on SMI-related outpatient visits according to sex, age, and SMI type (schizophrenia, bipolar disorders, and other SMIs) categories.

### 2.3. Statistical analysis

We used ITS analysis (19) to investigate whether the COVID-19 pandemic affected SMI-related outpatient visits. We used the following equation: *Y*_t_ = *ꞵ*_0_ + *ꞵ*_1_ * *T*_t_ + *ꞵ*_2_ * *X*_t_ + *ꞵ*_3_ * *P*_t_ + *S*_t_, where *Y*_t_ is the SMI-related outpatient department utilization at time t; *S*_t_ controls for the number of days in the month, the months with local outbreaks in Ningbo, and a seasonality factor (Fourier term); *T*_t_ is a continuous variable (from 1 to 54), indicating the number of months since the start of our study (January 2018); *X*_t_ is a dummy variable indicating the pre-pandemic period (coded 0, from January 2018 to December 2019) or the pandemic period (coded 1, from January 2020 to June 2022); and *P*_t_ is a continuous variable representing the number of months of the pandemic, coded 0 before the pandemic and (*T*_t_-24) during the pandemic. In this model, *ꞵ*_0_ represents the baseline number of outpatient visits at *T*_t_ = 0; *ꞵ*_1_ is the pre-pandemic slope of the number of outpatient visits by patients with SMI; *ꞵ*_2_ is whether the number of outpatient visits by patients with SMI changed immediately after the pandemic; and *ꞵ*_3_ is the difference in the number of outpatient visit slopes before and after the onset of the pandemic. In a sensitivity analysis, we analyzed the data only from January 2018 to December 2021, as several local outbreaks intensively occurred in Ningbo in 2022. We performed ITS regression analyses for sex and age subgroups. Age was categorized into the following five categories: <20, 20–39, 40–59, 60–79, and ≥80 years. Moreover, ITS analyses were performed to assess the effect of the COVID-19 pandemic on outpatient visits for individual SMI categories (schizophrenia, bipolar disorders, and other SMIs). We used Spearman’s correlation to estimate the relationship between the number of monthly outpatient visits and the number of incidents and accidents related to SMI. All analyses were conducted using the statistical package SAS version 9.4 (SAS Institute, Cary, NC, United States). Two-sided *p*-values less than 0.05 were considered statistically significant.

## 3. Results

**Figure 1:**
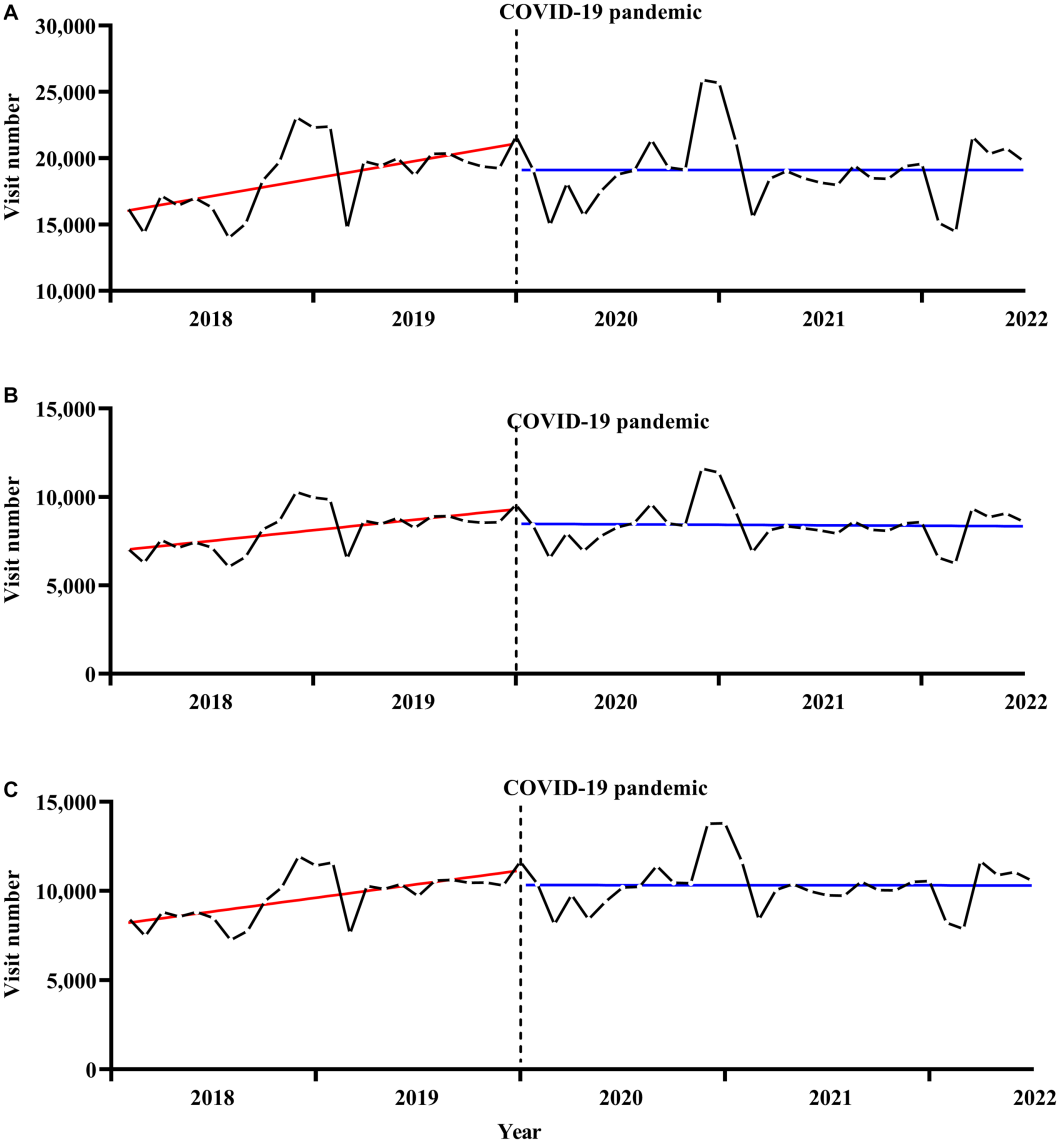
Secular trends in monthly outpatient visits for serious mental illness, **(A)** for total, **(B)** for males, **(C)** for females. The solid red and blue line represents the linear trend of SMI outpatient visits before and after the onset of the pandemic. The dotted black line represents the onset of the COVID-19 pandemic.

The number of SMI-related outpatient visits in Ningbo was 210,045 person-time in 2018, 235,667 in 2019, 237,889 in 2020, 224,225 in 2021, and 112,055 in the first 6 months of 2022 (Table 1). The details of the number of monthly outpatient visits overall and according to sex and age categories are shown in Supplementary Tables S2 and S3. Figures 1 2 illustrate the secular monthly trends in SMI-related outpatient visits overall and according to sex and age categories before (January 2018 to December 2020) and after (January 2020 to June 2022) the onset of the pandemic. The number of telemedicine visits each month ranged from 14 person-time units in June 2021 to 135 person-time units in April 2022 (Supplementary Table S2).

**Table 1:** Characteristic of outpatient visits from January 1, 2018 to June 30, 2022.

	2018	2019	2020	2021	2022^a^
Visit number	210,045	235,667	237,889	224,225	112,055
Sex					
Males	92,335	103,621	103,843	98,685	48,710
Females	108,602	123,723	126,375	121,590	60,276
Not available	9,108	8,323	4,371	3,950	3,069
Age					
<20 years	7,084	8,984	9,438	9,193	4,206
20–39 years	67,227	71,176	66,456	64,136	30,582
40–59 years	89,262	100,617	100,554	95,875	48,867
60–79 years	42,483	50,972	54,162	51,518	26,544
≥80 years	3,989	3,918	3,979	3,503	1856
Types of hospital					
Psychiatric specialist hospital	181,129	199,413	196,035	185,467	87,076
Others	28,916	36,254	38,554	38,758	24,979
Number of incidents and accidents	110	76	79	98	62

a The data from 1–6 months.

**Figure 2:**
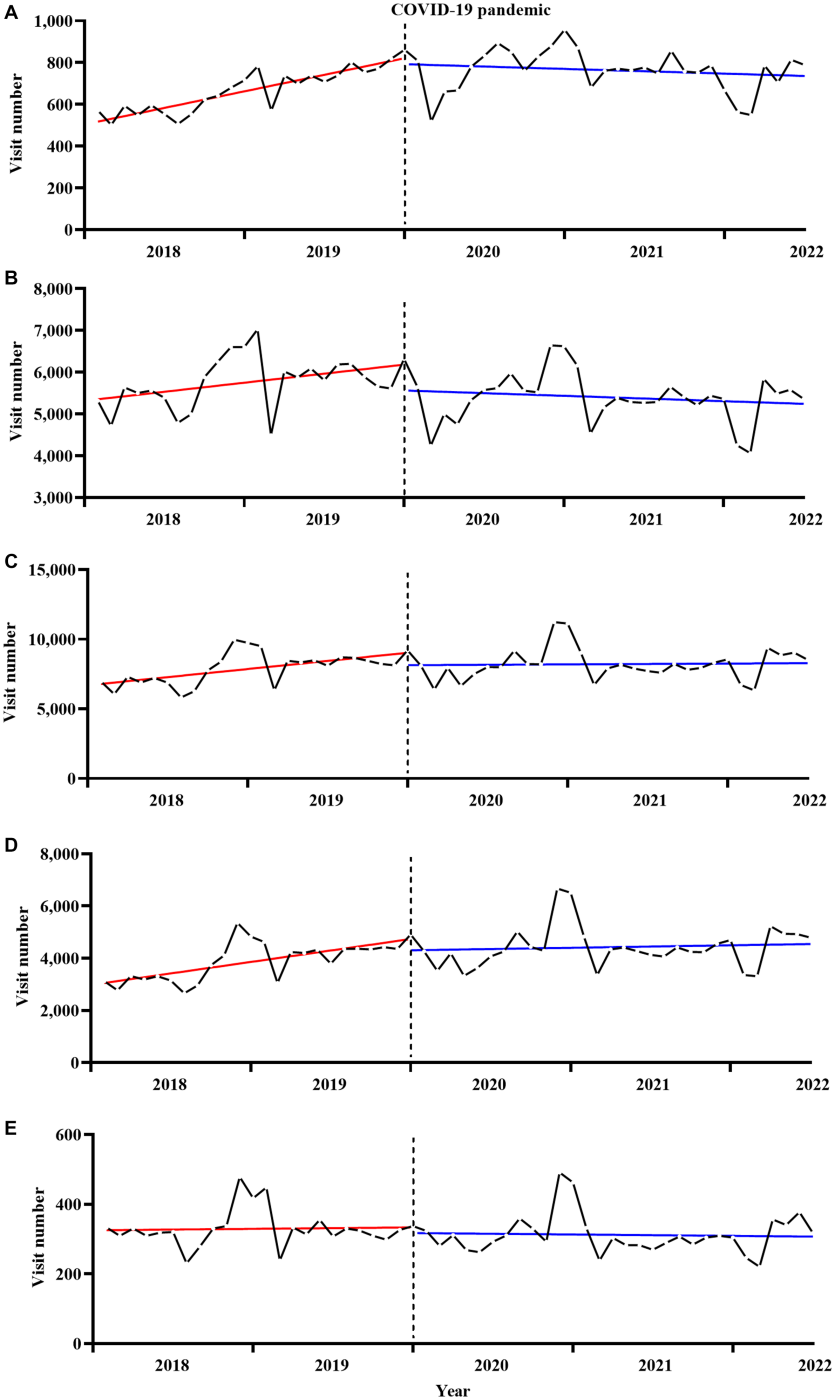
Secular trends in monthly outpatient visits for serious mental illness by age, **(A)** for <20 years, **(B)** for 20–39 years, **(C)** for 40–59 years, **(D)** for 60–79 years, **(E)** for ≥80 years. The solid red and blue line represents the linear trend of SMI outpatient visits before and after the onset of the pandemic. The dotted black line represents the onset of the COVID-19 pandemic.

Table 2 shows the results of the ITS regression analyses of the effect of the COVID-19 pandemic on outpatient visits for SMI. For the overall and sex-specific categories, no statistically significant immediate change in the number of outpatient visits was observed at the onset of the pandemic. However, the onset of the COVID-19 pandemic led to a significant and immediate decrease in the number of SMI-related outpatient visits per month in the 20–39 years age group [−612.4, 95% confidence interval (CI) (−1170.3 to −54.4), *p* < 0.05], but no statistically significant immediate changes were observed in the other age categories. A significant difference in the outpatient visit slopes before and after the onset of the pandemic was observed in the overall dataset [−175.6, 95% CI (−338.3 to −12.9), *p* < 0.05]. Moreover, all sex and age categories, except the 20–39 years age group [−33.6, 95% CI (−70.5 to 3.4), *p* > 0.05], showed statistically significant changes in slopes (all *p* < 0.05) before and after the onset of the pandemic. Sensitivity analyses were used to investigate the robustness of the results. The results of the sensitivity analyses did not substantially differ from the results of the main analyses (data not shown).

**Table 2:** Interrupted time series regression analyses of the impact of the COVID-19 pandemic on outpatient visits for serious mental illness during the pandemic period.

	Pre-pandemic trend^a^	Pandemic trend^b^	Level change^c^	Slopes change^d^
Total	200.4^**^ (68.1 to 332.6)	25.5 (−79.0 to 130.0)	−1966.2 (−4423.7 to 491.4)	−175.6^*^ (−338.3 to −12.9)
Sex				
Males	90.8^**^ (30.7 to 150.8)	6.6 (−40.5 to 53.7)	−803.2 (−1919.2 to 312.8)	−84.6^*^ (−158.5 to −10.7)
Females	117.1^**^ (48.9 to 185.4)	12.8 (−41.8 to 67.4)	−803.6 (−2071.9 to 464.8)	−104.8^*^ (−188.8 to −20.8)
Age				
<20 years	12.5^***^ (8.6 to 16.3)	−1.0 (−4.3 to 2.3)	−13.0 (−84.4 to 58.4)	−13.4^***^ (−18.2 to −8.8)
20–39 years	30.4^*^ (0.3 to 60.4)	−3.2 (−25.4 to 19.0)	−612.4^*^ (−1170.3 to −54.4)	−33.6 (−70.5 to 3.4)
40–59 years	88.9^**^ (30.0 to 147.8)	15.6 (−30.4 to 61.6)	−892.4 (−1986.6 to 201.9)	−73.6^*^ (−146.1 to −1.17)
60–79 years	68.7^**^ (29.1 to 108.3)	14.1 (−18.9 to 47.2)	−434.3 (−1170.2 to 301.7)	−54.8^*^ (−103.6 to −6.12)
≥80 years	0.01 (−3.3 to 3.3)	−0.07 (−2.5 to 2.4)	−14.1 (−75.3 to 47.1)	−0.1 (−4.2 to 4.0)

^*^p < 0.05, ^**^p < 0.01, ^***^p < 0.001.

a The pre-pandemic slope of outpatient visit before the onset of the pandemic.

b The pandemic slope of outpatient visit after the onset of the pandemic.

c The immediately change in the outpatient visit between before and after the onset of the pandemic.

d The difference in the outpatient visit slopes between before and after the onset of the pandemic.

**Figure 3:**
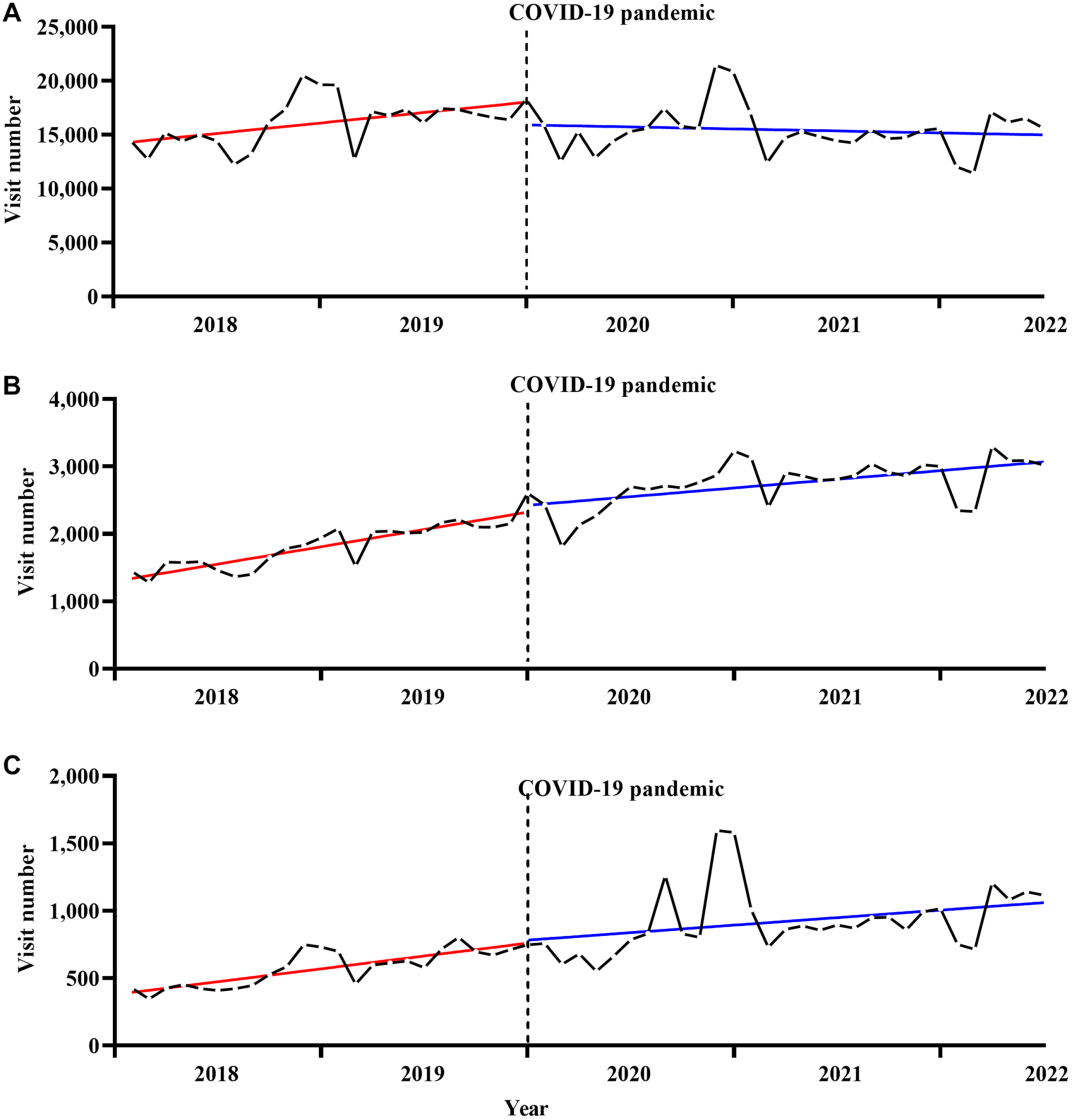
Secular trends in monthly outpatient visits for individual serious mental illness, **(A)** for schizophrenia, **(B)** for bipolar disorders, **(C)** for others serious mental illness. The solid red and blue line represents the linear trend of SMI outpatient visits before and after the onset of the pandemic. The dotted black line represents the onset of the COVID-19 pandemic.

Figure 3 illustrates the secular monthly trends in individual SMI outpatient visits before and after the onset of the pandemic. Table 3 shows the results of the ITS regression analyses of the effects of the COVID-19 pandemic on the number of outpatient visits for schizophrenia, bipolar disorders, and other SMIs. The onset of the COVID-19 pandemic led to a significant immediate decrease in the number of outpatient visits per month for schizophrenia [−2151.8, 95% CI (−4278.7 to −24.9), *p* < 0.05], but no statistically significant immediate change in the number of outpatient visits for bipolar disorders or other SMIs. Significant differences in the number of outpatient visit slopes before and after the onset of the pandemic were observed for schizophrenia [−153.3, 95% CI (−294.1 to −12.5)] and bipolar disorders [−16.8 (−31.0 to −2.6)]. However, no statistically significant changes in slopes were observed for other SMIs.

**Table 3:** Interrupted time series regression analyses of the impact of the COVID-19 pandemic on outpatient visits for individual serious mental illness during the pandemic period.

	Pre-pandemic trend^a^	Pandemic trend^b^	Level change^c^	Slopes change^d^
Schizophrenia	144.9^*^ (30.5 to 259.4)	−7.9 (−95.3 to 79.4)	−2151.8^*^ (−4278.7 to −24.9)	−153.3^*^ (−294.1 to −12.5)
Bipolar disorder	40.5^***^ (29.0 to 52.0)	23.9^***^ (14.1 to 33.7)	134.9 (−80.0 to 348.8)	−16.8^*^ (−31.0 to −2.6)
Others^e^	14.9^**^ (4.2 to 25.7)	9.5 (−0.7 to 19.7)	50.7 (−149.5 to 250.9)	−5.5 (−18.8 to 7.7)

^*^p < 0.05, ^**^p < 0.01, ^***^p < 0.001.

a The pre-pandemic slope of outpatient visit before the onset of the pandemic.

b The pandemic slope of outpatient visit after the onset of the pandemic.

c The immediately change in the outpatient visit between before and after the onset of the pandemic.

d The difference in the outpatient visit slopes between before and after the onset of the pandemic.

e Others included epileptic psychosis, schizoaffective disorders, intellectual disabilities and delusional disorders.

**Figure 4:**
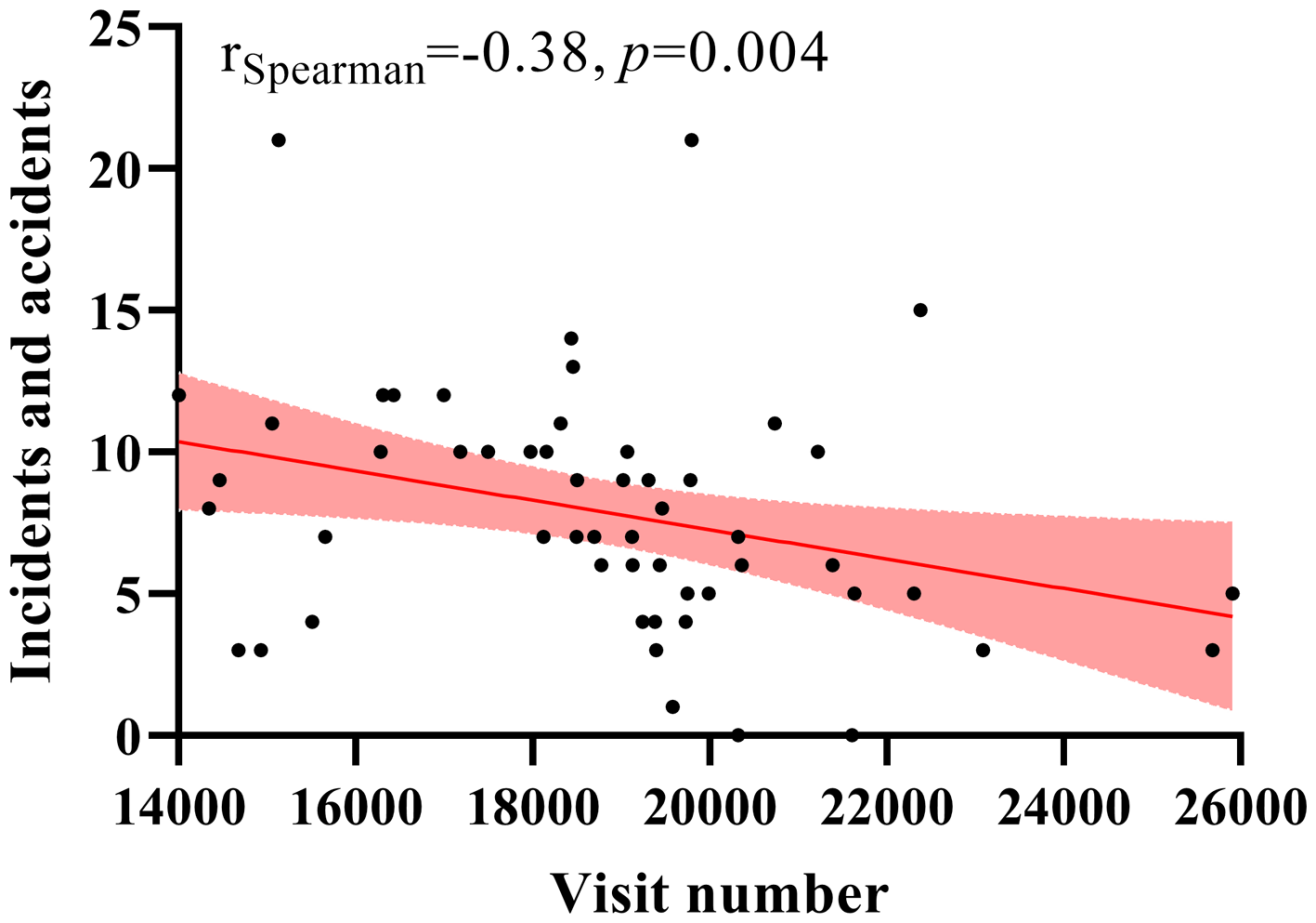
The relationship between monthly outpatient visits and incidents and accidents for serious mental illness.

Figure 4 shows the negative relationship between the number of monthly outpatient visits and the number of incidents and accidents due to SMI (*r* = −0.38, *p* < 0.05).

## 4. Discussion

To the best of our knowledge, this is the first study to examine the effect of the COVID-19 pandemic on SMI-related outpatient visits in China. Significant decreases in the number of outpatient visit slopes before and after the onset of the pandemic were observed for SMIs, especially schizophrenia.

Our results provide important information about reductions in the number of SMI-related outpatient visits during the COVID-19 pandemic, which is consistent with the results of other studies. In the United States, compared with 2019, the number of SMI-related outpatient visits decreased by 20.3% during the first 4 weeks of the pandemic (20). A study in South Africa showed that SMI-related outpatient consultations immediately decreased by 16% [odds ratio, 0.84; 95% CI (0.71–0.99)] after the introduction of lockdowns due to the COVID-19 pandemic (8). Moreover, in Southeast England, the number of weekly face-to-face community and outpatient contacts fell immediately by 70.4%, compared to the mean weekly number of face-to-face contacts prior to 2020 (18). A study from a Korean tertiary hospital reported a 13.3% reduction in the number of SMI-related outpatient care visits after the onset of the pandemic (7). In our study, the onset of the COVID-19 pandemic led to a significant immediate decrease in the number of outpatient visits per month by patients with schizophrenia [−2,151.8, 95% CI (−4278.7 to −24.9), *p* < 0.05]. Moreover, a prediction model used in a Korean study found that the actual number of patients with schizophrenia receiving outpatient treatment decreased by 1,605 compared to the expected number during the early COVID-19 outbreak (21). Although, there was no evidence from anywhere that the prevalence of SMI increased during the epidemic, the decreased number of SMI-related outpatient visits due to COVID-19 likely represents a large number of SMI patients that were not able to receive continuous treatment, rather than a decrease in the prevalence of SMI, given the evidence that most countries have reported increased rates of mental illness due to the pandemic (22, 23). Untreated SMI can result in an increase in the severity of symptoms and relapse of disease, and may even lead to social and financial problems or suicide and criminal behavior (24). The unmet need for mental health services may have long-term consequences for people with SMI.

Although the number of outpatient visit increased from 2019 to 2020, the number of visits did not reach the number of expected visits (expected increase of 200 visits/per month) without the pandemic. Also, there was decreasing but no statistically significant difference in the reduction of outpatient visits for SMI before and after the pandemic. The no significant immediate change in outpatient visits for SMI might be due to no measures being taken to close hospitals and stay-at-home in the early of outbreak, and only infected people or close contacts are quarantined. Thus, patients with SMI could seek medical help as usual in 2020, and the actual reduction of outpatient visits mainly resulted in the fear of infection.

Telemedicine visits, as an alternative to face-to-face visits, made up the majority of outpatient visits for patients with SMI in the initial weeks of the pandemic in the United States (20, 25). In fact, telepsychiatry outpatient care also ensures access to prescription medicines, as doctors were allowed to prescribe medication via telepsychiatry services (25). Therefore, continuous treatment was available for patients with SMI. However, telemedicine visits were rarely used in China, where this technology was still in its infancy (26). Telemedicine visits in Ningbo started in June 2021, and the maximum use was only 135 cases per month (Supplementary Table S2), which was not enough to replace the reduction in the number of outpatient visits. There are several reasons for the low use of telemedicine in Ningbo. As many studies have demonstrated, there are inequities in access to universal broadband and other critical technological infrastructure between different countries (27). Therefore, telemedicine visits are still in their infancy in China (28). Similarly, it is possible that, although telemedicine access was available to many patients with chronic illness, this mode of healthcare delivery may be less acceptable for patients with SMI (25, 29) due to their low self-care ability during daily living. In the future, telemedicine visits should be used on a large scale to cope with social isolation.

The following points may explain the reasons for the reductions in SMI-related outpatient visits. Social lockdowns and stay-at-home orders were implemented when the local COVID-19 outbreak occurred in Ningbo, and therefore, patients with SMI may have had difficulties in seeking medical care. Moreover, the fear of infection may have created a reluctance to visit hospitals in China (30), especially for patients with SMI and their families (31). Indeed, a meta analysis from 4 studies (no study from China) suggested individuals with SMI have experienced a greater risk of hospitalization and mortality due to COVID-19 than the general population (32). Individuals with SMI in China have been shown to be more sensitive to infection because of lack of insight due to decreased cognitive ability and awareness of self-care (33).

The strengths of our study were the large sample size and continuous longitudinal data from pre- and post-pandemic follow-ups. Moreover, this is the first study focusing on the effect of the COVID-19 pandemic on SMI-related outpatient visits in China. The ITS models used in our study used a quasi-experimental design, and we also adjusted for certain confounding variables (e.g., the seasonality factor). Finally, our results were shown to be robust in sensitivity and subgroup analyses.

However, our study had the following limitations. First, our data were limited to outpatient data. It is also important to determine the effect of the COVID-19 pandemic on hospitalization. Second, there were a lot of missing data regarding the sex of the patients. Thus, there may have been bias in the results of the effect of the COVID-19 pandemic according to sex subgroups. Third, we classified SMIs according to the ICD-10 codes from clinicians’ primary diagnoses recorded in the EHR system; thus, comorbid psychiatric disorders may have biased the results for individual SMIs. Besides, the present findings should not be generalized, as the results are only based on EHR data from Ningbo, a place without long-term social lockdowns due to the COVID-19 pandemic during the period of our study. Finally, sample selection bias should also be considered.

In conclusion, we found a decrease in the use of mental health services during the COVID-19 pandemic, suggesting that many patients were inadequately treated during this period. Therefore, mental health professionals should carefully monitor the ongoing treatment of patients with SMI and strengthen community mental health outpatient services in the face of the future epidemics. Careful planning and execution of preventive measures should be taken to minimize the negative effects of this pandemic. Telemedicine should be vigorously promoted by the government and hospitals.

## Data availability statement

The original contributions presented in the study are included in the article/Supplementary material, further inquiries can be directed to the corresponding author.

## Ethics statement

The studies involving human participants were reviewed and approved by the Ethics Committee of Ningbo Kangning Hospital, and permission was granted to analyze the data (NBKNYY-2022LC-41). Written informed consent to participate in this study was provided by the participants’ legal guardian/next of kin.

## Author contributions

GB and LL conceived and designed the study. RZ and HY compiled and prepared the study data. LL wrote the first draft. GB and YW reviewed the manuscript. All authors contributed to the article and approved the submitted version.

## Funding

This research was funded by Ningbo Medical and Health Brand Discipline (PPXK2018-08) and Ningbo Medical & Health Leading Academic Discipline Project (2022-F28).

## Conflict of interest

The authors declare that the research was conducted in the absence of any commercial or financial relationships that could be construed as a potential conflict of interest.

## Publisher’s note

All claims expressed in this article are solely those of the authors and do not necessarily represent those of their affiliated organizations, or those of the publisher, the editors and the reviewers. Any product that may be evaluated in this article, or claim that may be made by its manufacturer, is not guaranteed or endorsed by the publisher.
