# Supplementary material for: Effect of COVID-19 pandemic on serious mental illness-related outpatient department utilization in Ningbo, China: an interrupted time series analysis
Source: Front Psychiatry. 2023 Jul 13;14:1199408. doi: 10.3389/fpsyt.2023.1199408 (PMC10372427; doi:10.3389/fpsyt.2023.1199408)
Supplement: Supplementary file 1 [file Data_Sheet_1.docx]

Telemedicine

visits **Stable1**

Outpatient visit code

| Illness category | ICD-10-CM codes |
| --- | --- |
| Schizophrenia | F20.0-F20.9 |
| Bipolar disorders | F30.0-F30.9, F31.0-F31.9 |
| Others |  |
| Schizoaffective disorders | F25.0-F25.9 |
| Epileptic psychosis | F06.8 |
| Intellectual disabilities | F70.1, F70.8, F72.1, F72.8, F73.1, F73.8, F78.1, F78.8, F79.1, F79.8 |
| Delusional disorders | F22.0-F22.9 |

**Stable2**

Data of outpatient visits for serious mental disorders.

| Time | Schizop-hrenia | Bipolar disorders | Others^*^ | Serious mental disorders | Troubles and accidents | Telemedicine  visits | local outbreaks |
| --- | --- | --- | --- | --- | --- | --- | --- |
| 201801 | 14438 | 1445 | 428 | 16311 | 12 | - | 0 |
| 201802 | 12720 | 1279 | 346 | 14345 | 8 | - | 0 |
| 201803 | 15176 | 1581 | 425 | 17182 | 10 | - | 0 |
| 201804 | 14403 | 1574 | 453 | 16430 | 12 | - | 0 |
| 201805 | 14985 | 1588 | 422 | 16995 | 12 | - | 0 |
| 201806 | 14419 | 1457 | 409 | 16285 | 10 | - | 0 |
| 201807 | 12213 | 1364 | 423 | 14000 | 12 | - | 0 |
| 201808 | 13207 | 1401 | 446 | 15054 | 11 | - | 0 |
| 201809 | 16141 | 1647 | 528 | 18316 | 11 | - | 0 |
| 201810 | 17358 | 1786 | 586 | 19730 | 4 | - | 0 |
| 201811 | 20508 | 1832 | 749 | 23089 | 3 | - | 0 |
| 201812 | 19637 | 1940 | 731 | 22308 | 5 | - | 0 |
| 201901 | 19608 | 2079 | 699 | 22386 | 15 | - | 0 |
| 201902 | 12706 | 1517 | 455 | 14678 | 3 | - | 0 |
| 201903 | 17150 | 2033 | 597 | 19780 | 9 | - | 0 |
| 201904 | 16781 | 2042 | 613 | 19436 | 6 | - | 0 |
| 201905 | 17354 | 2011 | 627 | 19992 | 5 | - | 0 |
| 201906 | 16097 | 2025 | 577 | 18699 | 7 | - | 0 |
| 201907 | 17437 | 2168 | 715 | 20320 | 7 | - | 0 |
| 201908 | 17348 | 2212 | 803 | 20363 | 6 | - | 0 |
| 201909 | 16950 | 2101 | 696 | 19747 | 5 | - | 0 |
| 201910 | 16614 | 2099 | 670 | 19383 | 4 | - | 0 |
| 201911 | 16381 | 2152 | 712 | 19245 | 4 | - | 0 |
| 201912 | 18286 | 2605 | 747 | 21638 | 5 | - | 0 |
| 202001 | 15946 | 2427 | 758 | 19131 | 6 | - | 1 |
| 202002 | 12519 | 1807 | 602 | 14928 | 3 | - | 1 |
| 202003 | 15309 | 2131 | 680 | 18120 | 7 | - | 0 |
| 202004 | 12834 | 2270 | 551 | 15655 | 7 | - | 0 |
| 202005 | 14338 | 2495 | 662 | 17495 | 10 | - | 0 |
| 202006 | 15290 | 2701 | 785 | 18776 | 6 | - | 0 |
| 202007 | 15583 | 2659 | 830 | 19072 | 10 | - | 0 |
| 202008 | 17419 | 2710 | 1260 | 21389 | 6 | - | 0 |
| 202009 | 15798 | 2681 | 829 | 19308 | 9 | - | 0 |
| 202010 | 15554 | 2767 | 803 | 19124 | 7 | - | 0 |
| 202011 | 21441 | 2872 | 1596 | 25909 | 5 | - | 0 |
| 202012 | 20874 | 3227 | 1581 | 25682 | 3 | - | 0 |
| 202101 | 17085 | 3128 | 1008 | 21221 | 10 | - | 0 |
| 202102 | 12390 | 2394 | 729 | 15513 | 4 | - | 0 |
| 202103 | 14686 | 2908 | 862 | 18456 | 13 | - | 0 |
| 202104 | 15275 | 2860 | 887 | 19022 | 9 | - | 0 |
| 202105 | 14851 | 2794 | 855 | 18500 | 9 | - | 0 |
| 202106 | 14452 | 2811 | 894 | 18157 | 10 | 14 | 0 |
| 202107 | 14243 | 2864 | 871 | 17978 | 10 | 30 | 0 |
| 202108 | 15479 | 3039 | 948 | 19466 | 8 | 44 | 0 |
| 202109 | 14630 | 2912 | 953 | 18495 | 7 | 20 | 0 |
| 202110 | 14714 | 2863 | 858 | 18435 | 14 | 29 | 0 |
| 202111 | 15380 | 3026 | 993 | 19399 | 3 | 23 | 0 |
| 202112 | 15568 | 3000 | 1015 | 19583 | 1 | 125 | 1 |
| 202201 | 12031 | 2346 | 751 | 15128 | 21 | 52 | 1 |
| 202202 | 11420 | 2330 | 714 | 14464 | 9 | 36 | 0 |
| 202203 | 17107 | 3296 | 1205 | 21608 | 0 | 91 | 0 |
| 202204 | 16160 | 3084 | 1081 | 20325 | 0 | 135 | 1 |
| 202205 | 16507 | 3089 | 1140 | 20736 | 11 | 132 | 0 |
| 202206 | 15656 | 3022 | 1116 | 19794 | 21 | 100 | 0 |

^*^Others included epileptic psychosis, schizoaffective disorders, intellectual disabilities and delusional disorders.

**Stable3**

Data of outpatient visits for serious mental disorders in gender and age category.

| Time | Males | Females | <20 years | 20-39 years | 40-59 years | 60-79 years | ≥80 years |
| --- | --- | --- | --- | --- | --- | --- | --- |
| 201801 | 7112 | 8497 | 570 | 5317 | 6971 | 3120 | 333 |
| 201802 | 6268 | 7466 | 502 | 4713 | 6054 | 2767 | 309 |
| 201803 | 7568 | 8824 | 594 | 5634 | 7305 | 3319 | 330 |
| 201804 | 7113 | 8561 | 548 | 5502 | 6888 | 3183 | 309 |
| 201805 | 7434 | 8813 | 596 | 5559 | 7213 | 3309 | 318 |
| 201806 | 7141 | 8481 | 554 | 5391 | 6875 | 3145 | 320 |
| 201807 | 6042 | 7239 | 506 | 4786 | 5823 | 2655 | 230 |
| 201808 | 6608 | 7754 | 553 | 4994 | 6271 | 2958 | 278 |
| 201809 | 8151 | 9413 | 624 | 5881 | 7752 | 3729 | 330 |
| 201810 | 8643 | 10192 | 641 | 6248 | 8391 | 4113 | 337 |
| 201811 | 10281 | 11946 | 680 | 6601 | 9974 | 5356 | 478 |
| 201812 | 9974 | 11416 | 716 | 6601 | 9745 | 4829 | 417 |
| 201901 | 9859 | 11591 | 784 | 7027 | 9503 | 4624 | 448 |
| 201902 | 6498 | 7594 | 570 | 4491 | 6327 | 3052 | 238 |
| 201903 | 8651 | 10280 | 738 | 6018 | 8454 | 4237 | 333 |
| 201904 | 8468 | 10100 | 699 | 5874 | 8345 | 4205 | 313 |
| 201905 | 8801 | 10379 | 738 | 6089 | 8489 | 4321 | 355 |
| 201906 | 8239 | 9702 | 708 | 5806 | 8091 | 3787 | 307 |
| 201907 | 8891 | 10577 | 739 | 6185 | 8709 | 4357 | 330 |
| 201908 | 8922 | 10622 | 803 | 6203 | 8673 | 4360 | 324 |
| 201909 | 8636 | 10458 | 754 | 5893 | 8453 | 4338 | 309 |
| 201910 | 8538 | 10470 | 770 | 5661 | 8231 | 4423 | 298 |
| 201911 | 8563 | 10310 | 819 | 5610 | 8132 | 4358 | 326 |
| 201912 | 9555 | 11640 | 862 | 6319 | 9210 | 4910 | 337 |
| 202001 | 8390 | 10398 | 808 | 5642 | 8093 | 4265 | 323 |
| 202002 | 6536 | 8114 | 518 | 4237 | 6379 | 3514 | 280 |
| 202003 | 7957 | 9784 | 661 | 4997 | 7959 | 4192 | 311 |
| 202004 | 6929 | 8411 | 667 | 4747 | 6647 | 3326 | 268 |
| 202005 | 7770 | 9393 | 780 | 5322 | 7505 | 3626 | 262 |
| 202006 | 8276 | 10192 | 834 | 5571 | 8009 | 4070 | 292 |
| 202007 | 8520 | 10237 | 894 | 5618 | 7996 | 4254 | 310 |
| 202008 | 9611 | 11406 | 851 | 5976 | 9181 | 5022 | 359 |
| 202009 | 8507 | 10454 | 760 | 5560 | 8222 | 4437 | 329 |
| 202010 | 8367 | 10430 | 828 | 5517 | 8207 | 4280 | 292 |
| 202011 | 11603 | 13766 | 879 | 6645 | 11227 | 6667 | 491 |
| 202012 | 11377 | 13790 | 958 | 6624 | 11129 | 6509 | 462 |
| 202101 | 9190 | 11646 | 871 | 6160 | 9031 | 4827 | 332 |
| 202102 | 6864 | 8380 | 681 | 4528 | 6715 | 3351 | 238 |
| 202103 | 8116 | 10056 | 760 | 5151 | 7905 | 4337 | 303 |
| 202104 | 8340 | 10383 | 771 | 5385 | 8171 | 4413 | 282 |
| 202105 | 8225 | 9985 | 763 | 5286 | 7898 | 4271 | 282 |
| 202106 | 8107 | 9757 | 777 | 5269 | 7715 | 4127 | 269 |
| 202107 | 7937 | 9737 | 748 | 5285 | 7596 | 4060 | 289 |
| 202108 | 8607 | 10517 | 857 | 5653 | 8234 | 4415 | 307 |
| 202109 | 8140 | 10039 | 758 | 5406 | 7806 | 4241 | 284 |
| 202110 | 8083 | 10029 | 752 | 5211 | 7938 | 4230 | 304 |
| 202111 | 8500 | 10501 | 788 | 5440 | 8313 | 4549 | 309 |
| 202112 | 8576 | 10560 | 667 | 5362 | 8553 | 4697 | 304 |
| 202201 | 6571 | 8216 | 563 | 4251 | 6713 | 3355 | 246 |
| 202202 | 6245 | 7870 | 549 | 4061 | 6326 | 3308 | 220 |
| 202203 | 9349 | 11665 | 786 | 5843 | 9395 | 5228 | 356 |
| 202204 | 8858 | 10878 | 705 | 5484 | 8858 | 4938 | 340 |
| 202205 | 9073 | 11062 | 814 | 5581 | 9038 | 4926 | 377 |
| 202206 | 8614 | 10585 | 789 | 5362 | 8537 | 4789 | 317 |
